# Characterization of a novel family VIII esterase EstM2 from soil metagenome capable of hydrolyzing estrogenic phthalates

**DOI:** 10.1186/s12934-020-01336-x

**Published:** 2020-03-24

**Authors:** Jayita Sarkar, Arindam Dutta, Piyali Pal Chowdhury, Joydeep Chakraborty, Tapan K. Dutta

**Affiliations:** Department of Microbiology, Bose Institute, P-1/12 CIT Scheme VII M, Kolkata, 700054 India

**Keywords:** Esterase, Metagenome, Unculturable, *β*-lactamase, Biocatalyst, Phthalate

## Abstract

**Background:**

Microbes are rich sources of enzymes and esterases are one of the most important classes of enzymes because of their potential for application in the field of food, agriculture, pharmaceuticals and bioremediation. Due to limitations in their cultivation, only a small fraction of the complex microbial communities can be cultured from natural habitats. Thus to explore the catalytic potential of uncultured organisms, the metagenomic approach has turned out to be an effective alternative method for direct mining of enzymes of interest. Based on activity-based screening method, an esterase-positive clone was obtained from metagenomic libraries.

**Results:**

Functional screening of a soil metagenomic fosmid library, followed by transposon mutagenesis led to the identification of a 1179 bp esterase gene, *estM2*, that encodes a 392 amino acids long protein (EstM2) with a translated molecular weight of 43.12 kDa. Overproduction, purification and biochemical characterization of the recombinant protein demonstrated carboxylesterase activity towards short-chain fatty acyl esters with optimal activity for *p*-nitrophenyl butyrate at pH 8.0 and 37 °C. Amino acid sequence analysis and subsequent phylogenetic analysis suggested that EstM2 belongs to the family VIII esterases that bear modest similarities to class C *β*-lactamases. EstM2 possessed the conserved S-x-x-K motif of class C *β*-lactamases but did not exhibit *β*-lactamase activity. Guided by molecular docking analysis, EstM2 was shown to hydrolyze a wide range of di- and monoesters of alkyl-, aryl- and benzyl-substituted phthalates. Thus, EstM2 displays an atypical hydrolytic potential of biotechnological significance within family VIII esterases.

**Conclusions:**

This study has led to the discovery of a new member of family VIII esterases. To the best of our knowledge, this is the first phthalate hydrolase (EstM2), isolated from a soil metagenomic library that belongs to a family possessing *β*-lactamase like catalytic triad. Based on its catalytic potential towards hydrolysis of both phthalate diesters and phthalate monoesters, this enzyme may find use to counter the growing pollution caused by phthalate-based plasticizers in diverse geological environment and in other aspects of biotechnological applications.

## Background

**Fig. 1:**
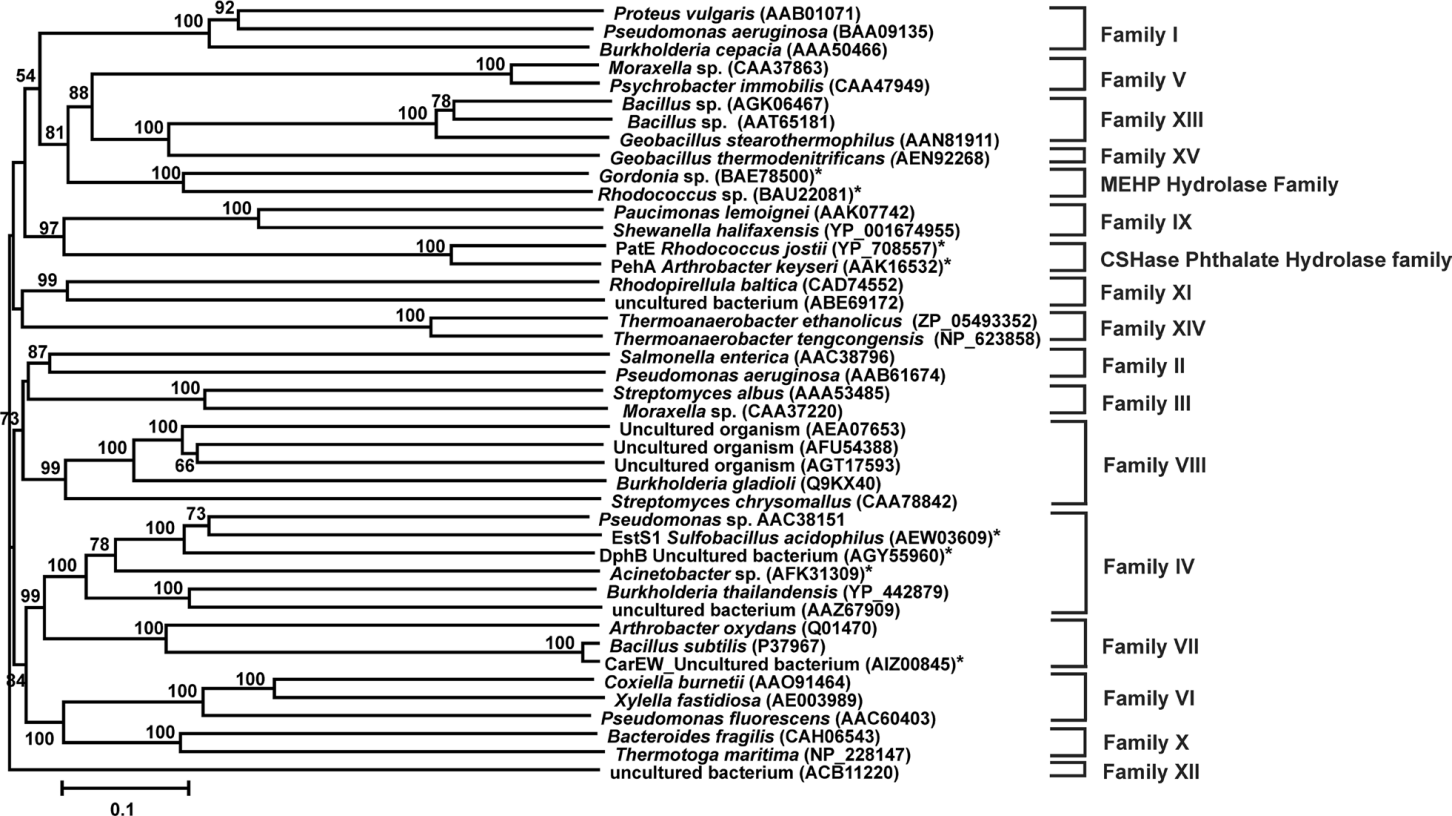
Phylogenetic tree based on the protein sequences of bacterial lipolytic enzymes belonging to different enzyme families. Numbers at the nodes indicate the levels of bootstrap support based on neighbour joining analysis of 100 resampled data sets. Bootstrap values below 50% are not shown. The scale bar represents 0.1 substitutions per nucleotide position. GenBank accession numbers of the sequences are indicated within parentheses. Phthalate ester hydrolases are denoted with an asterisk

Bacterial carboxylesterases (EC 3.1.1.1) are ubiquitous in nature and are known to catalyze the hydrolysis of ester bonds of a structurally diverse array of compounds. Most members of this enzyme group are serine hydrolases belonging to the characteristic α/β hydrolase fold superfamily [1]. These enzymes usually possess a catalytic triad composed of Ser, His and Asp (or Glu) residues and a well conserved G-x-S-x-G motif around the active site nucleophilic serine [1]. The catalytic serine residue has also been identified in several carboxylesterases possessing either a G-D-S-L [2] or a *β*-lactamase S-x-x-K motif [3]. Based on sequence identity, biochemical properties and conserved sequence motifs, microbial lipolytic enzymes were traditionally classified into eight different families by Arpigny and Jaeger [4]. Nevertheless, with the discovery of novel lipolytic enzymes [5, 6], there is a continuous expansion of the original classification scheme as shown in Fig. 1. Recently, sequence-based comparison studies have further updated the existing classification of bacterial lipolytic enzymes [7].

Among them, esterases belonging to family VIII are unique because they show sequence similarity with class C *β*-lactamases and penicillin binding proteins [3]. This family of proteins is structurally distinct where the position of the nucleophilic serine that acts as the active site residue occurs in the conserved N-terminal S-x-x-K motif. On the other hand, in the majority of lipolytic enzyme families, active site serine is present in the classical G-x-S-x-G pentapeptide motif or in the G-D-S-L motif [8]. Although most of the family VIII esterases do not exhibit *β*-lactamase activity on standard *β*-lactam substrates [8–11], EstC [12], EstM-N1/EstM-N2 [13], EstU1 [14] and SbLip1 [15] are the few family VIII members that were found to display *β*-lactamase activity against chromogenic *β*-lactam substrate nitrocefin. Among these, EstU1 was reported to exhibit *β*-lactamase activity towards first generation *β*-lactam antibiotics such as cephalosporins, cephaloridine, cephalothin, and cefazolin [14]. Recently, Jeon et al. [16] reported another novel family VIII esterase (EstSTR1), which hydrolyzes third and fourth generation cephalosporins, cefotaxime and cefepime in addition to the first generation antibiotics. It is believed that the conserved Ser and Lys residues of the S-x-x-K motif, and another conserved Tyr residue, forms the catalytic triad for ester hydrolysis in family VIII esterases, similar to that of class C *β*-lactamase enzymes, capable of hydrolyzing *β*-lactam ring [17, 18].

Lipolytic enzymes, esterases and lipases have a wide range of biotechnological applications in food, perfume and pharmaceutical industries because of their broad substrate specificities, regio- and stereoselectivity, remarkable stability in organic solvents, and no requirement for cofactors [19]. Due to up-growing demands for lipolytic enzymes in industry, researchers are keen to screen novel lipolytic enzymes from terrestrial and marine environmental samples by metagenomic approaches [20–23]. Apart from industrial application, esterases are the key enzymes in the degradation of estrogenic phthalate esters, one of the most abundant groups of man-made environmental pollutants, accounting for a number of potential causes of human health problems including developmental and testicular toxicity as well as exhibiting antiandrogenic, teratogenic, and carcinogenic effects [24, 25]. Although there are numerous reports on the metabolism of phthalate mono- and diesters in bacteria [26–30], genetic information on phthalate esterases/hydrolases that catalyzes the first step in the degradation of phthalate ester is quite limited. As far as metabolism of phthalate esters is concerned, there are only a few reports on the purified dialkyl phthalate esterase enzymes, which are capable of specifically hydrolyzing only one of the ester bonds of the diester to generate the corresponding monoalkyl phthalate esters [28, 30–35]. On the other hand, there are only limited reports of specific monoalkyl phthalate esterases that hydrolyze monoalkyl phthalates to furnish phthalic acid but are incapable of hydrolyzing phthalate diesters [5, 36–40]. Recently, another esterase isolated from naphthalene-enriched soil community, was shown to hydrolyze various polyaromatic esters besides dimethyl phthalate [41]. Nonetheless, to the best of our knowledge, there are only two reports describing the degradation of both phthalate mono- and diesters by a single phthalate esterase [42, 43]. Sequence analysis of reported monoalkyl and dialkyl phthalate esterases revealed that these enzymes belong to different lipolytic enzyme families and harbor family-specific signature motifs in their sequences [5, 32–34, 36, 38, 42, 44] (Fig. 1). However, till date, no phthalate hydrolase was reported that belonged to Family VIII esterase.

In the present study, we report the screening and biochemical characterization of a novel family VIII esterase isolated from a municipal waste-contaminated soil metagenomic library. In addition, we also shed insight into the three dimensional structural model of the esterase followed by docking to evaluate the in silico substrate specificity of the esterase towards estrogenic phthalates and subsequent experimental validation on the hydrolytic potential of the enzyme.

## Methods

### Bacterial strains, plasmids and culture conditions

Strains and plasmids used in this study are listed in (Additional file 1: Table S1). *E. coli* was grown at 37 °C in Luria-Bertani (LB) medium. Antibiotics (100 μg mL^−1^ ampicillin, 25 μg mL^−1^ kanamycin or 12.5 μg mL^−1^ chloramphenicol at final concentration) and Isopropyl-β-*D*-thiogalactopyranoside (IPTG, 1 mM at final concentration) were added to the culture medium whenever required.

### Purification of metagenomic DNA and construction of fosmid library

Metagenomic DNA was extracted from soil according to a method described by Zhou et al. [45]. Contaminated humic materials present in extracted crude DNA sample (100 μg) were resolved by long gel electrophoresis and pure DNA was obtained by electroelution of nucleic acid from the corresponding gel band. Purified DNA was then concentrated using a concentrator (MWCO 10,000, Sartorius, Germany). Purified DNA (2.5 μg) was end-repaired and 5′-phosphorylated followed by ligation with the Cloning-Ready CopyControl™ HTP pCC2Fos™ fosmid vector provided with CopyControl™ HTP fosmid library production kit (Epicentre, USA) according to the manufacturer’s instructions. The ligated DNA was packaged on EPI300™-T1R Phage T1-resistant *E. coli* and plated onto LB agar plates containing chloramphenicol (12.5 μg mL^−1^) to construct a metagenomic library. The clones were allowed to grow overnight. Finally, the fosmid clones were replica plated in LB agar plates containing chloramphenicol.

### Functional screening for esterase activity

Fosmid clones were streaked onto LB agar plates supplemented with tributyrin (1%) and chloramphenicol. Clones showing lipolytic activity were identified based on the formation of clear zones around colonies after incubation for 48 h at 37 °C.

### Identification and sequencing of esterase gene

Fosmid DNA was isolated from clones using the FosmidMAX DNA purification kit (Epicentre, USA) and the isolated fosmids were used for in vitro transposon mutagenesis using the commercially available EZ-Tn5 <KAN-2> insertion kit (Epicentre, USA). Mutants exhibiting no zone formation on tributyrin-agar plates were sequenced using the primers supplied with the kit to identify the inactivated gene. Nucleotide sequencing was performed with an ABI 3100 automated sequencer using a BigDye Terminator kit (Perkin-Elmer Applied Biosystems, Foster City, CA). DNA sequence analysis, database search, and gene characterization were carried out at the National Center for Biotechnology Information (NCBI; http://www.ncbi.nlm.nih.gov/) using web-based programs and resources.

### Computational analyses

Homologues of the lipolytic enzyme were identified using Blastx v2.2.25 [46] and the sequences were retrieved from NCBI. Multiple sequence alignments of protein sequences and construction of phylogenetic tree by neighbor-joining method were performed using Clustalx v1.81 [47]. The trees were visualized and manipulated using TreeExplorer v2.12. The SignalP ver. 3.0 software was used for the identification of potential signal peptides [48].

For homology modeling, suitable template selection for the lipolytic enzyme was performed by identifying closest homologue(s) in the Brookhaven Protein Data Bank (PDB) using Blastp [47]. A three-dimensional model was constructed based on sequence alignment by spatial restrictions technique using Modeller 9v7 [49]. Depending on bound ligand, it appeared that alternative rotameric states do exist for several protein side chains, therefore, the protein was modelled with the template structure in complex with one of its natural substrate. The models were finally checked using Prochek, Verify3D and VADAR [50–52]. Catalytic pocket of the modeled structure was predicted with CASTp [53] using a solvent probe of radius 1.4 Å and was compared with known structures.

Molecular docking was carried out using incremental construction method as implemented in FlexX. The NCBI PubChem database (http://pubchem.ncbi.nlm.nih.gov/) was used to obtain co-ordinates of the ligands (described in Table 2). For docking experiments using FlexX program, standard parameters as implemented in the 2.1.3 release of the LeadIT (BioSolveIT, GmbH, Germany) were used for iterative growing and subsequent scoring of the target-bound conformations and orientations (here, called solutions) and the active site was defined as the collection of amino acids enclosed within a 10 Å radius sphere centered on the reference ligand [54]. For each ligand, the best solution was chosen from 100 independent docking simulations according to correct orientation of active site residues towards the substrate as well as high docking score and low clash score. The solution was finally stored as a mol2 file. Another set of docking analyses were performed using grid based method as implemented in Autodock 4.0 to corroborate the findings obtained by FlexX. The pdb files of ligands and proteins were converted into pdbqt files using AutoDock Tools 1.5.6. AutoGrid4 was used to define a grid box centred on the C_α_ coordinates of Ser68 with the dimensions of 50 by 50 by 50 grid points and a grid spacing of 0.375A^0^. AutoDock4 was used to dock each ligand for 50 times using genetic algorithm where the number of energy evaluations was set to maximum. Docked poses of each ligand were grouped into spatially distinct clusters using 2A^0^ root-mean-square-deviations (RMSDs) cut-off. The binding-poses were analyzed using AutoDockTools-1.5.6 and were compared with the FlexX output.

### Over-expression and purification of recombinant esterase

The full length gene was amplified from fosmid DNA using Platinum *Taq* DNA Polymerase (Invitrogen, Carlsbad, CA), cloned in the expression vector pET-28a, and transformed into *E. coli* BL21(DE3) to generate clone *E*. *coli* BL21-pETESTM2. The primers used were 5′-GGAATTCCATATGAATGCTGTTCCTTCCCTTGC-3′ (forward; estf1) and 5′-CGCGGATCCTCGGTCAGCAGTGTGATGAAG-3′ (reverse; estr1) with restriction enzyme sites (underlined) for NdeI and BamHI, respectively, designed to generate an N-terminal His-tag of the recombinant enzyme. For protein overexpression, *E*. *coli* BL21-pETESTM2 was grown in 500 ml LB medium with 50 μg mL^−1^ kanamycin at 37 °C until an OD_600_ of 0.5 was reached. Then IPTG was added to a final concentration of 1 mM and the cells were further incubated at 37 °C for 3 h. The cells were then centrifuged and the pellet was washed twice with equal volume of 50 mM phosphate buffer (pH 7.0). Finally, the pellet was resuspended in 10 ml of Lysis buffer [NaH_2_PO_4_ (50 mM), NaCl (300 mM), imidazole (10 mM) and Glycerol (10%)] and loaded into a pre‐cooled French press, Constant Cell Disruption System, One Shot 182 Model (Constant System Ltd., United Kingdom) fitted with a 8.0 ml cell and lysed at 16,000 psi for one cycle. Cell‐free extracts were obtained by centrifuging the cell homogenates at 20,000×*g* for 30 min at 4 °C. The expressed recombinant protein was purified from the crude cell-free extract by a rapid, one-step procedure using nickel-nitrilotriacetic acid (Ni–NTA) resin (Qiagen, Hilden, Germany) followed by overnight dialysis against 100 mM phosphate buffer saline (PBS) to remove imidazole. Protein concentration was determined using Bradford protein assay method, with bovine serum albumin (BSA) as standard [55]. Purity of the protein was checked by SDS-PAGE, and protein bands were visualized by Coomassie Brilliant Blue R-250. The same protein was also subjected to non-denaturing 10% polyacrylamide gel electrophoresis for activity staining as described previously [56].

### Biochemical characterization of esterase

Substrate specificities were determined by using *p*-nitrophenyl esters (*p*-NP ester) with different aliphatic side chains: C2 (acetate), C4 (butyrate), C6 (hexanoate), C8 (octanoate), C10 (decanoate), C12 (laurate), C14 (myristate) and C16 (palmitate). The standard assay mixture contained 1 mM *p*-NP ester, 1% acetonitrile and 1 μl (4.6 ng) of purified esterase in a total volume of 1 ml of 50 mM Tris-HCl buffer (pH 8.0). The reaction mixture was incubated for 8 min and terminated by the addition of 20 μl 10% SDS. The enzymatic activity was measured by monitoring absorbance at 405 nm, indicating the production of *p*-nitrophenol (extinction coefficient, 17000 M^−1^ cm^−1^) against a substrate-free blank, using a Cary 100 Bio UV–visible spectrophotometer (Varian Australia).

The optimum temperature of the enzyme reaction was determined using *p*-NP butyrate as substrate at various temperatures ranging from 16 to 65 °C, while its thermostability was determined by incubating reaction mixtures at 25, 35, 45 and 55 °C and measuring the residual activity of the enzyme at different time period of incubation up to 1 h. The optimum pH was determined as described above over a pH range of 5.0 to 10.0, using the following buffer systems (50 mM): citrate buffer (pH 5.0–6.0), sodium phosphate buffer (pH 6.0–7.5), Tris-HCl (pH 7.5–9.0) and glycine–NaOH (pH 9.0–10.0). Similarly, effects of various metal ions (Mn^2+^, Mg^2+^, Ca^2+^, Cu^2+^, Fe^2+^, Co^2+^, Hg^2+^, Zn^2+^and Ni^2+^) and enzyme inhibitors [N-ethylmaleimide, diethylpyrocarbonate (DEPC), *p*-chloromercuribenzoic acid (*p*-CMB) and phenylmethylsulfonyl fluoride (PMSF)] on enzyme activity were determined by incubating metal ion or inhibitor individually with the purified enzyme under the reaction conditions as mentioned above for 1 h, and subsequently measuring the residual enzyme activity. Concentrations of metal ions and inhibitor used were 5 mM and 1 mM, respectively. In addition, effect of organic solvents on esterase activity was examined using methanol, ethanol, acetone, chloroform, acetonitrile and isopropanol at final concentrations of 1%, 5% or 15% as described above. The activity of the enzyme without additive was defined as 100%. Measurements were performed in triplicate. For kinetic analyses, *K*_m_ and V_max_ were calculated by using a non-linear curve fitting program, Graphpad Prism 4, based on the Michaelis–Menten equation whereas catalytic efficiency (*k*_cat_/*K*_m_) of the purified esterase were calculated by using the values of *K*_m_, V_max_ and the molecular weight of the native protein.

### Assay of phthalate hydrolase activity

For analysis of phthalate hydrolase activity, 1 mM of each substrate (dissolved in methanol) and 0.25 μg of purified protein were added to a final volume of 1 ml Tris–HCl buffer (50 mM, pH 8.0). Each sample was incubated at 37 °C for 1 h and the reaction mixture was extracted twice with equal volume of ethyl acetate. After extraction, ethyl acetate was evaporated under reduced pressure and the metabolites were dissolved in a small volume of methanol. The concentration of residual substrate was determined by HPLC using a Shimadzu model LC20-AT pump system (Shimadzu Corp., Kyoto, Japan) equipped with a diode array model SIL-M20A detector and an analytical Phenomenex C18 reverse-phase column (Phenomenex Inc., Torrance, CA) attached to a model SIL-20A auto sampler. The biodegraded products were eluted with a programmed gradient of methanol and potassium phosphate buffer (50 mM, pH 5.2) for 60 min at a flow rate of 1.0 mL min^−1^ and detected at 254 nm along with diode array analysis. Metabolites were identified by comparing their retention times as well as UV–visible spectra with those of the pure, control compounds analyzed under the same set of conditions. Kinetic parameters were calculated for each substrate from nonlinear regression data analysis against a concentration range of 0.1–10 mM. One unit of enzyme activity is defined as the amount of enzyme required to degrade 1.0 μmol of substrate per minute at 25 °C. Three independent determinations were performed for each kinetic constant.

### Nucleotide sequence accession number

The nucleotide sequence for *estM2* retrieved from metagenome has been deposited in DDBJ/EMBL/GenBank under the Accession Number KP113669.

## Results and discussion

### Cloning and sequence analysis of gene responsible for lipolytic activity

A metagenomic library consisting of 8174 fosmid clones was constructed using purified metagenomic DNA isolated from a municipal waste-contaminated soil sample (Dhapa, Kolkata, India; 22.5602°N, 88.4421°E). Screening of the library for lipolytic activity on LB agar plate containing 1% (v/v) tributyrin and chloramphenicol resulted in the identification of a positive clone, designated as *E. coli* EPI300-fosEstM2 showing clear zone around the colony (Additional file 1: Fig. S1). Since sequence analysis cannot identify an obvious candidate gene from sequenced ORFs present within a 35–40 kb insert of a recombinant fosmid, transposon mutagenesis was performed to disrupt the expression of the specific esterase followed by identification of the gene. In vitro transposon mutagenesis of the fosmid pCC2FOSESTM2 (Additional file 1: Table S1) identified a 1179 bp gene, responsible for the lipolytic activity and was designated as ‘*estM2*′ (Additional file 1: Fig. S2). The gene showed a high GC content (68.28%) encoding the protein EstM2 comprising of 392 amino acids. No signal sequence was predicted in EstM2. EstM2 exhibited a high level of sequence identity (> 90%) with several uncharacterized esterases (annotated as class C *β*-lactamases) from both cultured and uncultured bacteria. Among biochemically characterized esterases, EstM2 showed maximum identities of 56.39% and 55.68% with EstBL and Lip8, from *Burkholderia cepacia* UWC10 (GenBank: AAX78516) and *Pseudomonas aeruginosa* LST-03 (GenBank: BAD69792), respectively. On the other hand, EstM2 showed a mere 20% identity with biochemically characterized class C *β*-lactamase, AmpC from *Enterobacter cloacae* P99.

### Phylogenetic relationships of EstM2

**Fig. 2:**
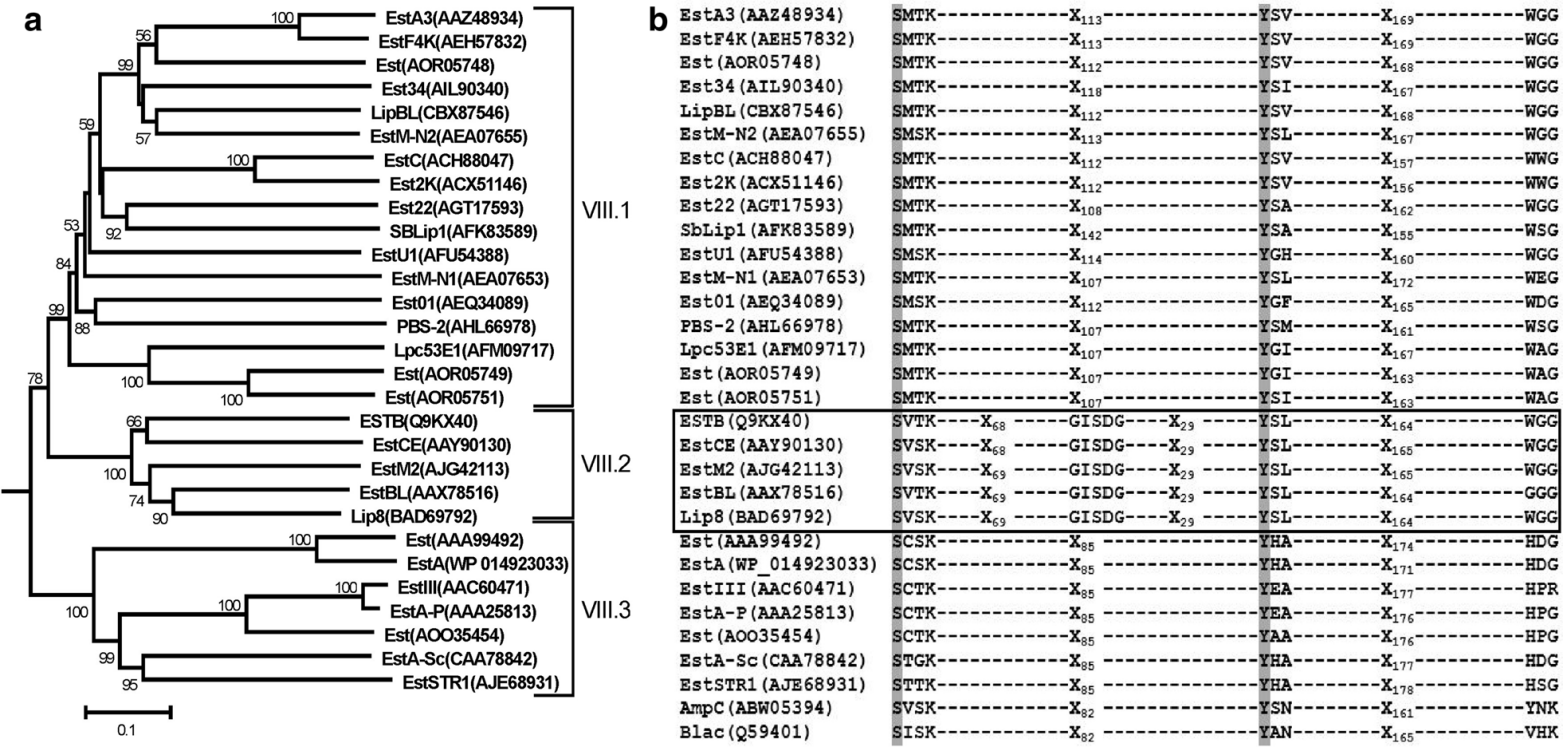
**a** Phylogenetic analysis of EstM2 with reported family VIII esterases. Numbers at the nodes indicate the levels of bootstrap support based on neighbor joining analysis of 100 resampled data sets. Bootstrap values below 50% are not shown. The scale bar represents 0.1 substitutions per nucleotide position. GenBank accession numbers are indicated within parentheses. AmpC, a class C *β*-lactamase from *Aeromonas enteropelogenes* (ABW05394) was used as an outgroup. **b** Multiple sequence alignment of EstM2 and other family VIII carboxylesterases showing the conserved sequence motifs. Amino acid residues responsible for the formation of catalytic triad for ester bond hydrolysis are shaded. Protein sequences with classical pentapeptide G-x-S-x-G motif are shown within a box. Alignment of two class C *β*-lactamases from *Aeromonas enteropelogenes* (AmpC, ABW05394) and *Enterobacter cloacae* (Blac, Q59401) respectively were also shown for sequence comparisons

Phylogenetic analysis of EstM2 with other homologous lipolytic enzymes revealed that it belongs to the family VIII of esterases. Figure 2a shows the phylogenetic tree constructed using reported family VIII esterases including EstM2. The analysis revealed the presence of three distinct clusters within the tree suggesting the presence of three evolutionarily diversified clades in family VIII esterases which we named as subfamily VIII.1, VIII.2 and VIII.3 respectively. Multiple sequence alignment of EstM2 and other family VIII esterases identified the presence of a S^68^-V-S-K^71^motif, compatible with the N-terminal S-x-x-K motif (Fig. 2B) conserved in class C *β*-lactamases [14] and penicillin binding proteins [3]. The role of the nucleophilic serine within the S-x-x-K motif in family VIII esterase and class C *β*-lactamase for the hydrolysis of respective substrates had already been confirmed by site-directed mutagenesis [8–13, 16, 57, 58]. This agrees with the fact that the classical pentapeptide esterase motif (G-x-S-x-G), reported to harbor the catalytic serine in a number of esterase families [1] is absent in most of the family VIII esterases. But EstM2, along with other family VIII members like Lip8, EstCE, EstB and EstBL, bear the pentapeptide motif at the far N-terminal part of the protein (G^141^-V-S-D-G^145^ for EstM2) (Fig. 2b). On the other hand, EstC, Est2K and LipBL were shown to harbor the G-x-S-x-G motif (which could not be included in Fig. 2b) at the C-terminal end [8, 10, 12]. A careful inspection of the multiple sequence alignment as well as the phylogenetic tree indicated that esterases bearing the far N-terminal pentapeptide signature motif form a discrete subgroup within family VIII esterases which was referred as subfamily VIII.2 (Fig. 2a). However none of the members belonging to subfamily VIII.2 showed sequence conservation for the other two conserved residues viz., Asp/Glu and His that completes the catalytic triad (as revealed in other classical esterases) [1]. Site directed mutagenesis of EstB and LipBL, the closest neighbor of EstM2 which also harbors the similar G-x-S-x-G esterase motif revealed that the Ser residue present in the G-x-S-x-G motif is not crucial for esterase activity but may alter the biochemical properties and substrate specificities of the esterase [3, 8]. On the other hand, the multiple sequence alignment showed the presence of a conserved Tyr residue in all family VIII esterases (Tyr^175^ in EstM2), which is the first residue of the conserved Y-x-x motif in family VIII esterases. This residue, along with the Ser and Lys residues of the S-x-x-K motif, forms the catalytic triad for ester hydrolysis in family VIII esterases [11, 14]. In addition, family VIII esterases also shows the WGG (subfamily VIII.1 and subfamily VIII.2) or the H–x-x (subfamily VIII.3) conserved motifs at the C-terminal end of the protein sequence which is considered as the remnant of the KTG motif found in class C *β*-lactamases (Fig. 2B). Recently, Lee et al. [59] also suggested a similar sub-classification for family VIII esterases where the presence of two distinct subfamilies (VIII.1 and VIII.2) has been proposed with the closest homologs of EstM2 being included in the subfamily VIII.1.

### Heterologous expression and purification of recombinant EstM2

To investigate the functional role of EstM2 in ester bond hydrolysis, the *estM2* gene was overexpressed and the His-tagged recombinant protein was purified from cell-free extract of *E. coli* BL21cells, harboring pETESTM2. The purified protein appeared as a single band on SDS-PAGE with a molecular mass of around 43 kDa (Additional file 1: Fig. S3A). The estimated molecular mass of 43 kDa is in accordance with the theoretical molecular mass of 43.12 kDa which corresponds well with the molecular mass of other family VIII esterases [12]. Activity staining of purified native protein EstM2 using α-naphthyl acetate and Fast blue BB in polyacrylamide gel revealed an esterase-active band, confirming the functional role of EstM2 as ester-bond hydrolyzing enzyme (Additional file 1: Fig. S3B).

### Biochemical properties of esterase

**Fig. 3:**
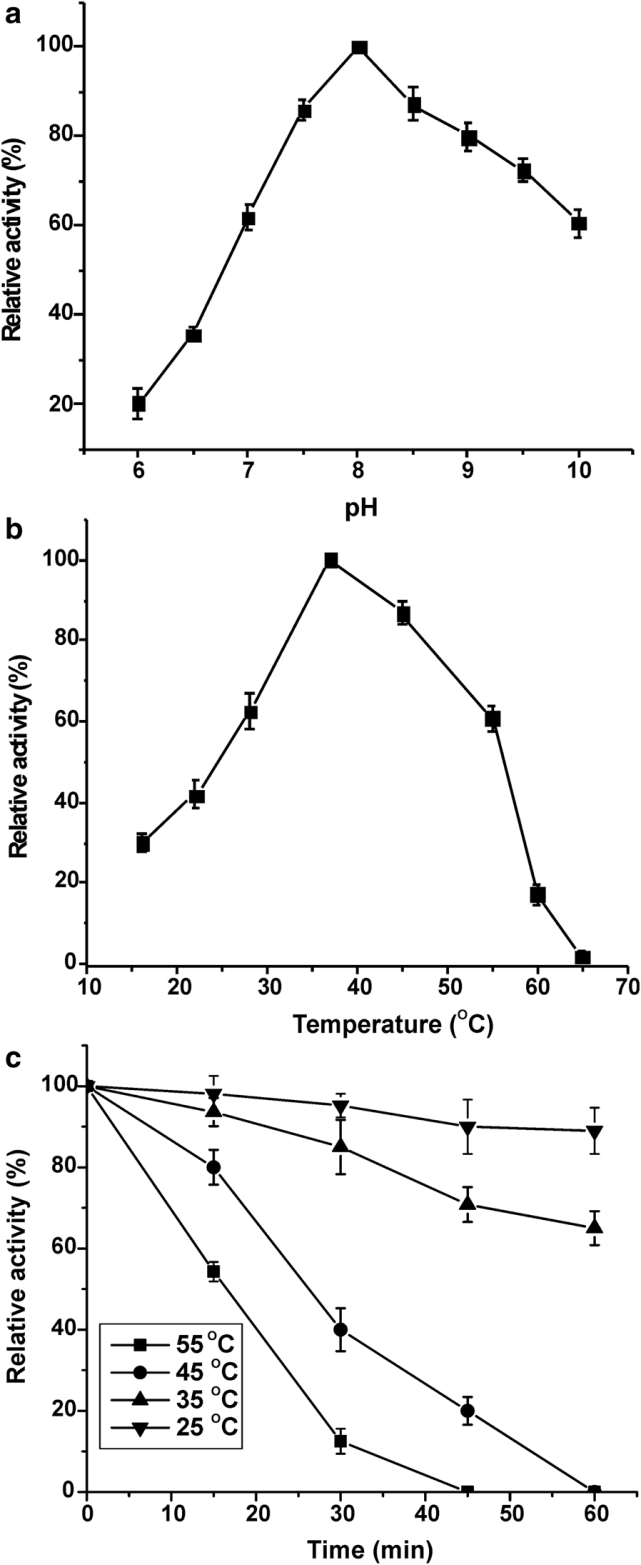
Plots showing relative activity of EstM2 at different pH (**a**), temperature (**b**) and on thermostability (**c**)

Assays for substrate specificity using *p*-nitrophenyl esters revealed that EstM2 can hydrolyze esters with short chain fatty acids (from C2 to C6) (Additional file 1: Table S2) exhibiting the highest activity against *p*-nitrophenyl butyrate followed by *p*-nitrophenyl acetate and *p*-nitrophenyl hexanoate with specific activities of 641.03 U mg^−1^, 520.8 U mg^−1^ and 57.5 U mg^−1^, respectively. The activity of EstM2 tested under buffered conditions over the pH range of 5 to 10, displayed high levels of activity under neutral to slightly alkaline conditions, with the highest activity at pH 8.0 (Fig. 3a). Moreover, we observed that EstM2 was active over a wide temperature range, from 16 to 55 °C, with maximum activity at 37 °C and a complete loss of activity at 65 °C (Fig. 3b). Regarding thermostability, enzyme EstM2 remained stable at 25 and 35 °C for at least 60 min without any major loss of activity. However, incubation at higher temperatures (45 and 55 °C) for more than 45 min resulted in a rapid inactivation of the enzyme (Fig. 3c).

EstM2 was tolerant to commonly used solvents viz., methanol, ethanol and isopropanol retaining over 90% of its activity even in the presence of 15% (v/v) of the solvents. However, the enzyme was most sensitive to chloroform, losing around 80% of its activity in the presence 5% of the solvent. Similarly, more than half of its activity was lost in the presence of 5% of acetone and acetonitrile (Additional file 1: Table S3). It has been observed that divalent cations inhibited the esterase activity to different extents (Table 1). Moreover, PMSF, a serine residue-specific inhibitor at a concentration of 1 mM inhibited 97% of the activity (Table 1), indicating that Ser68 residue is possibly responsible for the catalytic activity [14]. As anticipated, cysteine residue-specific inhibitor *p*-CMB showed no inhibition on EstM2, a cysteine-free polypeptide.

**Table 1:** Effect of metal ion and inhibitor on the activity of EstM2

Metal ion/Inhibitor	Relative activity (%)
None	100 ± 3.8
Mn^2+^	55.8 ± 2.2
Mg^2+^	48.1 ± 2.1
Ca^2+^	38.7 ± 1.8
Cu^2+^	10.8 ± 0.8
Fe^2+^	20.3 ± 1.1
Co^2+^	20.5 ± 1.0
Ni^2+^	35.7 ± 1.6
Zn^2+^	109.4 ± 4.4
Hg^2+^	52.83 ± 2.6
PMSF	3.6 ± 0.2
DEPC	41.46 ± 1.8
*p*-CMB	105 ± 4.6
N-ethylmaleimide	104.24 ± 3.9

The relative activities are given as a percentage of the activity in the absence of metal ion/inhibitor. The data is represented as an average of three replicates

When *E. coli* BL21-pETESTM2 cells were cultured in LB medium supplemented with penicillin G or cephalexin, no growth was observed suggesting that EstM2 is not able to hydrolyse standard *β*-lactam antibiotics, although its protein sequence showed similarities to various class C *β*-lactamases. Also, no spectral change of nitrocefin (a chromogenic *β*-lactam substrate) was observed in presence of purified EstM2 (data not shown), confirming that EstM2 does not have any *β*-lactamase activity unlike a number of other members of subfamily VIII.1 esterases [12–14, 60]. It may be mentioned here that none of the members of subfamily VIII.2 are reported to have *β*-lactamase activity, whereas EstC, EstM-N1, EstM-N2 and SBLip1, belonging to the subfamily VIII.1, show *β*-lactamase activity only towards nitrocefin. EstU1 is the first characterized family VIII esterase belonging to the subfamily VIII.1 which shows *β*-lactamase activity and hydrolyses the amide bond of first generation *β*-lactam antibiotics [14]. Recently, Jeon et al. [16] reported a novel family VIII esterase from a soil metagenomic library which is capable of hydrolysing third as well as fourth generation cephalosporins, cefotaxime and cefepime, in addition to first generation cephalosporins and cephalothin. Est22, on the other hand, can hydrolyze the ester bond of *β*-lactam antibiotics [60] showing traditional esterase activity but lacks *β*-lactam hydrolase activity.

### Molecular three-dimensional modelling and docking analyses

A search for homologues of the derived protein sequence of EstM2 in the Brookhaven Protein Data Bank (PDB) yielded a close resemblance (49.86% identity over 367 amino acids) with that of the esterase EstB from *Burkholderia gladioli* (PDB: 1CI9). Also, the catalytic residues Ser68, Lys71, and Tyr175 in EstM2 are conserved with Ser75, Lys78, and Tyr181 in EstB, which are crucial for ester hydrolysis [11]. Using 1CI9 as template, a structural model of EstM2 has been constructed. The tertiary conformation of the catalytic domain of the EstM2, as observed from Prochek, showed a good energy plot having an optimum mean force potential.

**Fig. 4:**
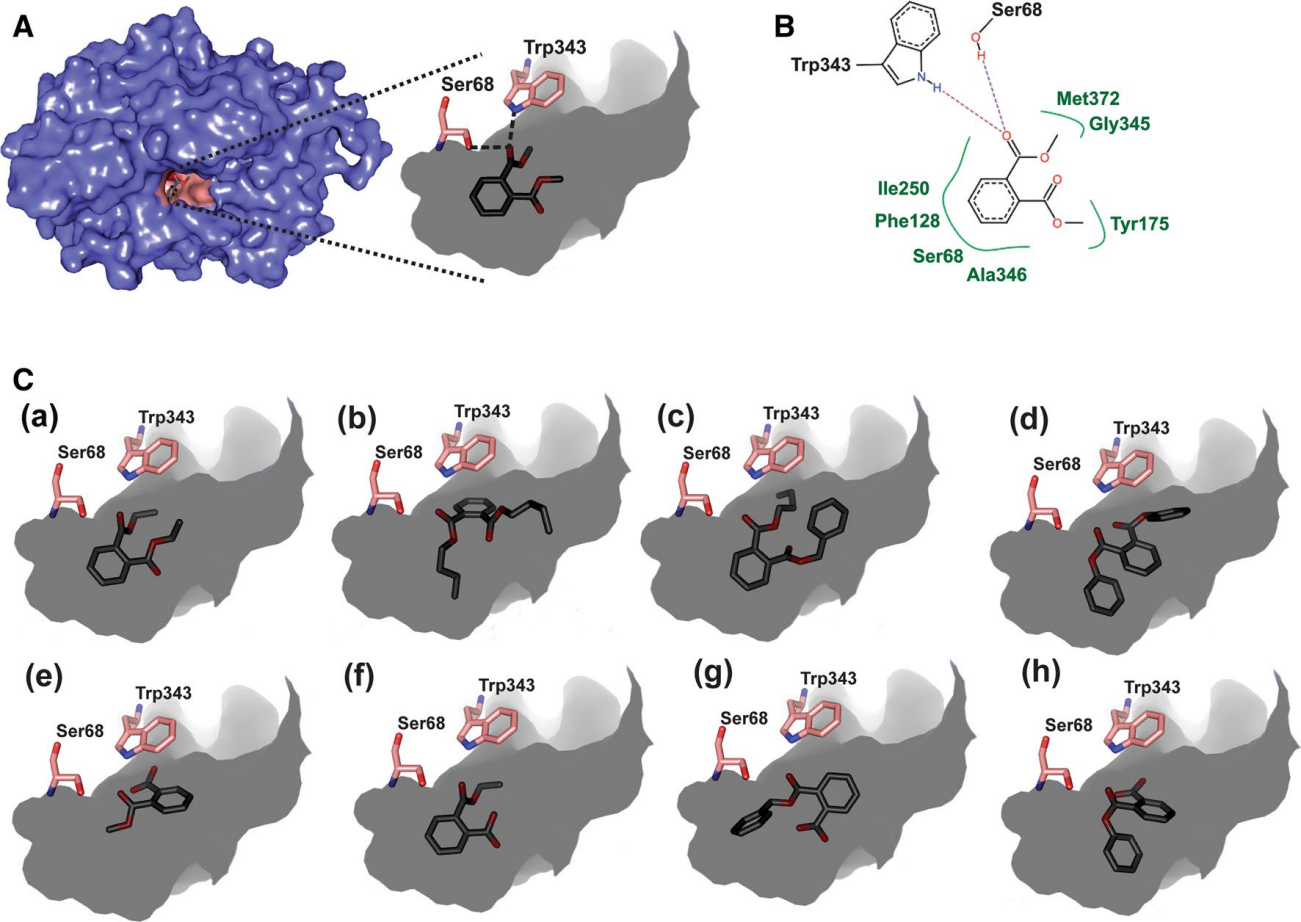
**A** Surface topology of EstM2 showing the binding of dimethyl phthalate within the catalytic pocket (pink) via electrostatic interaction with active site residues Ser68 and Trp343 based on docking analysis; **B** Lucid view showing hydrophobic interactions of dimethyl phthalate with different residues at the catalytic pocket of EstM2; **C** Alignment of other phthalate esters within the catalytic pocket of EstM2, as obtained from docking studies: diethyl phthalate (a), di-*n*-butyl phthalate (b), butyl benzyl phthalate (c), diphenyl phthalate (d), monomethyl phthalate (e), monoethyl phthalate (f), monobenzyl phthalate (g) and monophenyl phthalate (h)

Synthetic plasticizers (phthalate diesters) are amongst the ingredients of various types of plastic household products that are largely found in municipal waste materials [61]. Since the library was constructed using metagenomic DNA of municipal waste-contaminated soil, several phthalate diesters and monoesters, viz. dimethyl phthalate (DMP), diethyl phthalate (DEP), di-*n*-butyl phthalate (DBP), butyl benzyl phthalate (BBP), diphenyl phthalate (DPP), di-*n*-octyl phthalate (DOP), di-2-ethylhexyl phthalate (DEHP), monomethyl phthalate (MMP), monoethyl phthalate (MEP), monobutyl phthalate (MBP), monobenzyl phthalate (MBzP) and monophenyl phthalate (MPP) were considered as substrates for docking with EstM2. Molecular docking was carried out using FlexX to evaluate the possible binding site of phthalate esters in EstM2. Figure 4a shows the active site of EstM2 docked with DMP whereas Fig. 4b, a computer generated output from FlexX analysis shows a lucid view of possible interactions of DMP with different residues at the binding pocket of the receptor. Docking studies predicted that DMP aligns within the catalytic pocket in a favourable position to interact electrostatically with EstM2 through the active site residues Ser68 and Trp343 (Fig. 4a) further corroborating the role of Ser68 of the S-x-x-K motif as the active site residue. Trp343, on the other hand, is the first residue of the conserved WGG motif found in family VIII.1 and VIII.2 esterases and is found to be equally important for phthalate ester hydrolysis. In addition, the residues Phe128, Tyr175, Ile250, Gly345, Ala346 and Met372 were predicted to interact with the substrate through hydrophobic interactions (Fig. 4b). Similar docked poses for other ligands considered in this study are shown in Fig. 4c. Furthermore, the cluster analysis of the binding poses of the ligands generated by Autodock revealed that in most of the cases the ligands participate in electrostatic interaction with Ser68 and Trp343, which is consistent with the FlexX results. The scores obtained from docking of EstM2 with di- and mono-substituted phthalate esters are listed in Supplementary information (Additional file 1: Table S4). It may be mentioned here that due to high clash scores as found in FlexX, BEHP was not found to align favourably within the catalytic pocket.

### Phthalate hydrolase activity

Based on the above docking results, we tested the ability of EstM2 to hydrolyze both phthalate di- and monoesters individually as substrates. Hydrolyzed products were identified by HPLC analysis based on retention time, UV–visible spectrum and co-elution profile of reference compounds. HPLC elution profile of hydrolyzed products of DBP (Additional file 1: Fig. S4) is shown as a representative of EstM2-mediated hydrolysis of phthalate esters employed in this study. The observed retention times for BBP, DPP, DBP, DEP, DMP, MBzP, MBP, MPP, MEP, MMP and phthalic acid were found to be 29.22, 28.10, 26.82, 25.18, 24.25, 19.86, 18.40, 17.41, 9.82, 9.22 and 5.45 respectively under the HPLC conditions used. Kinetic analysis revealed that apart from *p*-nitrophenyl esters, the enzyme could utilize most of the phthalate esters tested. Table 2 shows the *K*_m_, V_max_ and catalytic efficiency (*k*_cat_/*K*_m_) values for each substrate. It was observed that in most of the cases, affinity of the enzyme for substrate (*K*_m_) decreased as the length of the side chain of both phthalate di- and monoesters increases. The hydrolytic potential of EstM2 towards phthalate esters was in the order DBP > BBP > DPP > DEP > DMP > MBP > MBzP > MPP > MEP > MMP (Table 2). The catalytic efficiency of EstM2 was found to be in the range of 15–90 mM ^−1^ s^−1^ which is close to that observed for DBP hydrolase isolated from *Camelimonas* sp. [25]. Recently, a cold-active phthalate hydrolase (DphB) isolated from a metagenomic library derived from biofilm of a wastewater treatment plant has been shown to have relatively high catalytic efficiency towards dialkyl phthalate esters [29] However, the substrate range for DphB is quite limited, metabolizing only dipropyl phthalate (DPrP), DBP and DPP as compared to EstM2 which can hydrolyze a range of mono- and dialkyl phthalate esters (Table 2). The catalytic efficiencies obtained for esterase CN1E1 [41], diisobutyl phthalate esterase (CarEW) [42] and DBP hydrolase [33], on the other hand, were found to be quite low as compared to EstM2. Kinetic studies also indicated that phthalate mono esters are hydrolyzed at a much slower rate than the corresponding phthalate diester which agrees well with kinetic studies of non-enzymatic base-catalyzed hydrolysis of mono- and dialkyl phthalate ester [62]. Monoalkyl phthalates (MAP) are difficult to be hydrolyzed by carboxyl esterases obtained from rat and human liver [63]. The slower rate of hydrolysis of MAP can be explained by the presence of carboxylate anion near the ester bond which imparts strong electrostatic repulsion toward the catalytic nucleophile attacking ester bond. However, it is suggested that a positively charged residue present in the catalytic site of monoalkyl phthalate esterase may help in counteracting the negative charge of the carboxylate anion [37].

**Table 2:** Kinetic constants of EstM2

Phthalate ester	*V*_max_ (µmol mg ^−1^min^−1^)	*K*_m_ (mM)	*k*_cat_/*K*_m_ (mM^−1^ s^−1^)
Dimethyl phthalate (DMP)	42.14 ± 3.3	1.02 ± 0.08	29.63 ± 2.3
Diethyl phthalate (DEP)	58.98 ± 3.8	0.80 ± 0.05	52.84 ± 3.4
Di-*n*-butyl phthalate (DBP)	72.87 ± 4.4	0.62 ± 0.04	84.23 ± 5.1
Butyl benzyl phthalate (BBP)	57.58 ± 3.4	0.64 ± 0.04	64.48 ± 3.8
Diphenyl phthalate (DPP)	45.10 ± 3.0	0.59 ± 0.04	54.78 ± 3.6
Monobenzyl phthalate (MBzP)	43.78 ± 3.2	1.12 ± 0.08	28.02 ± 2.0
Monobutyl phthalate (MBP)	56.67 ± 3.9	1.26 ± 0.08	32.24 ± 2.2
Monomethyl phthalate (MMP)	41.28 ± 3.2	1.95 ± 0.15	15.17 ± 1.2
Monoethyl phthalate (MEP)	54.21 ± 4.0	1.73 ± 0.13	22.46 ± 1.6
Monophenyl phthalate (MPP)	37.59 ± 2.8	1.06 ± 0.08	25.42 ± 1.9
*p*-nitro phenyl acetate (C_2_)	747.9 ± 18.8	0.41 ± 0.01	1292.5 ± 32.5
*p*-nitro phenyl butyrate (C_4_)	905.0 ± 21.2	0.44 ± 0.01	1462.1 ± 34.2
*p*-nitro phenyl hexanoate (C_6_)	71.39 ± 4.2	0.28 ± 0.02	182.78 ± 10.7

The data is represented as an average of three replicates

## Conclusion

In the present study, activity-based screening of metagenomic library has led to the discovery of a new member of family VIII esterases, EstM2. Subsequent purification and biochemical characterization of the recombinant protein showed the highest substrate specificity for short chain fatty acyl esters (C2–C6). Despite being highly similar to type C *β-*lactamases, EstM2 did not show any *β-*lactamase activity. Interestingly, the esterase is capable of hydrolyzing both phthalate diesters and phthalate monoesters and is active over a wide range of temperature and pH without any cofactor requirement. Although phthalate hydrolases have long been isolated from cultured microbial sources and typically possess the catalytic serine within the consensus G-x-S-x-G motif [36–38, 40], to the best of our knowledge, EstM2 is the first phthalate hydrolase, isolated from a soil metagenomic library that belongs to family VIII esterases possessing a *β*-lactamase like catalytic triad for phthalate ester hydrolysis. It is believed that structural modification of the enzyme by site-directed mutagenesis may further enhance the catalytic performance of EstM2 towards various estrogenic phthalates and may find use to counter the growing pollution caused by phthalate-based plasticizers in diverse geological environment and in other aspects of biotechnological applications.

## Supplementary information

**Additional file 1: Table S**1. Strains and plasmids used in this study. **Table S2.** Specificity of EstM2 against p-nitrophenyl ester substrates. **Table S3.** Effect of solvent on the activity of EstM2^a,b^. **Table S4.** Scores obtained from docking of EstM2 with di- and mono-substituted phthalate esters^a^. **Fig. S1.** Screening for lipolytic enzyme activity on 1% (v/v) tributyrin agar plate. Inset shows formation of clear zone by fosmid clone, *E. coli* EPI300-fosEstM2. **Fig. S2.** Mutants of esterase-positive clone by in vitro transposon mutagenesis using the commercially available EZ-Tn5 <KAN-2> insertion kit (Epicentre, USA). Mutants did not exhibit zone formation on tributyrin agar plate. **Fig. S3.** (A)SDS-PAGE of the purified EstM2 protein. M, molecular size markers; lane 1, whole-cell extracts before induction; lane 2, whole-cell extracts after induction; lane 3, EstM2 purified by Ni-nitrilotriacetic acid column (denatured); (B) Activity staining of EstM2 purified by Ni-nitrilotriacetic acid column (non-denatured). Arrows indicate position of the band corresponding to EstM2. **Fig. S4.** HPLC profile of di-n-butyl phthalate and its metabolic intermediates in the reaction mixture containing 1 mM of substrate (dissolved in methanol) and 0.25 μg of purified protein (EstM2) in a final volume of 1 ml Tris-HCl buffer (50 mM, pH 8.0), incubated for 1 h. Insets, UV-visible spectra of peaks obtained with diode array analysis. I, di-n-butyl phthalate; II, mono-n-butyl phthalate, III, phthalic acid.

## Data Availability

All data generated or analyzed during this study are included in this published article.
