# Supplementary material for: Characterization of a novel family VIII esterase EstM2 from soil metagenome capable of hydrolyzing estrogenic phthalates
Source: Microb Cell Fact. 2020 Mar 24;19:77. doi: 10.1186/s12934-020-01336-x (PMC7092541; doi:10.1186/s12934-020-01336-x)
Supplement: Supplementary file 1 — Additional file 1: Table S1. Strains and plasmids used in this study. Table S2. Specificity of EstM2 against p-nitrophenyl ester substrates. Table S3. Effect of solvent on the activity of EstM2a,b. Table S4. Scores obtained from docking of EstM2 with di- and mono-substituted phthalate estersa. Fig. S1. Screening for lipolytic enzyme activity on 1% (v/v) tributyrin agar plate. Inset shows formation of clear zone by fosmid clone, E. coli EPI300-fosEstM2. Fig. S2. Mutants of esterase-positive clone by in vitro transposon mutagenesis using the commercially available EZ-Tn5 insertion kit (Epicentre, USA). Mutants did not exhibit zone formation on tributyrin agar plate. Fig. S3. (A)SDS-PAGE of the purified EstM2 protein. M, molecular size markers; lane 1, whole-cell extracts before induction; lane 2, whole-cell extracts after induction; lane 3, EstM2 purified by Ni-nitrilotriacetic acid column (denatured); (B) Activity staining of EstM2 purified by Ni-nitrilotriacetic acid column (non-denatured). Arrows indicate position of the band corresponding to EstM2. Fig. S4. HPLC profile of di-n-butyl phthalate and its metabolic intermediates in the reaction mixture containing 1 mM of substrate (dissolved in methanol) and 0.25 μg of purified protein (EstM2) in a final volume of 1 ml Tris-HCl buffer (50 mM, pH 8.0), incubated for 1 h. Insets, UV-visible spectra of peaks obtained with diode array analysis. I, di-n-butyl phthalate; II, mono-n-butyl phthalate, III, phthalic acid. [file 12934_2020_1336_MOESM1_ESM.pdf]

Supplementary Information

Article Title: **Characterization of a novel family VIII esterase EstM2 from soil metagenome capable of hydrolyzing estrogenic phthalates**

Journal: Microbial Cell Factories

Authors: Jayita Sarkar, Arindam Dutta, Piyali Pal Chowdhury, Joydeep Chakraborty and Tapan K. Dutta

Affiliation: Department of Microbiology, Bose Institute, Kolkata 700054, India

E-mail address of corresponding author: tapan@jcbose.ac.in

Additional File 1

Table S1 Strains and plasmids used in this study

	**Features**	**Source**
Strains		
EPI300™-T1R Phage T1-Resistant *E. coli* Plating strain	*F⁻ mcrA Δ(mrr-hsdRMS-mcrBC) Φ80dlacZΔM15 ΔlacX74 recA1 endA1 araD139 Δ(ara, leu)7697 galUgalK λ⁻ rpsL* (*Str*^{R}) *nupGtrfAtonA*	Epicentre Biotechnologies (Madison, USA)
E. coli BL21 (DE3)	*F⁻, ompT, hsdSB (rB-, mB-), dcm, gal, lon, λ(DE3)*	Novagen
E. coli EPI300-fosEstM2	Positive clone carrying the lipolytic gene	This study
E. coli BL21-pETESTM2	*E. coli* BL21 (DE3) cells harboring the plasmid pETESTM2	This study
Plasmids		
CopyControl™ pCC2FOS™	Chloramphenicol-resistant, linearized at the unique *Eco*72I site, P1 *lox*P, dephosphorylated, T7 RNA polymerase promoter flanking the cloning site, *E. coli* F factor-based partitioning and copy number regulation system	Epicentre Biotechnologies (Madison, USA)
pET28a	Expression vector; Km^r	Novagen
pCC2FOSESTM2	Derived from pCC2FOS contains ~38kb metagenomic DNA as insert	This study
pETESTM2	pET28a carrying amplified HindIII -NdeI fragment containing lipolytic gene (EstM2)	This study

Table S2 Specificity of EstM2 against *p*-nitrophenyl ester substrates

Substrate	Specific activity[a] (U/mg)	Relative Activity[b] (%)
p-Nitrophenyl acetate (C2)	520.8±8.2	81.24
p-Nitrophenyl butyrate (C4)	641.03±7.6	100
p-Nitrophenylhexanoate (C6)	57.5±0.9	8.97
p-Nitrophenyloctanoate (C8)	ND[c]	ND[c]
p-Nitrophenyldecanoate (C10)	ND[c]	ND[c]

[a]Specific activity is defined as μmol substrate hydrolysed min^{-1} $(mg\ protein)^{-1}$ under standard assay conditions.

[b]For calculation of relative activity, specific activity against *p*-nitrophenyl butyrate (C4) was set as 100%

[c] Not detected

Table S3 Effect of solvent on the activity of EstM2[a,b]

Solvent	Relative activity (for 1% solvent)	Relative activity (for 5% solvent)	Relative activity (for 15% solvent)
Methanol	110 ± 4.7	102 ± 3.1	90.9 ± 2.1
Ethanol	204.9 ± 5.2	263.8 ± 7.2	220.1 ± 4.9
Isopropanol	104 ± 4.1	120.8 ± 4.6	97.5 ± 2.5
Acetone	70.8 ± 3.5	55.5 ± 1.7	6.9 ± 0.4
Acetonitrile	97.2 ± 2.7	50.7 ± 1.2	9.8 ± 0.3
Chloroform	45.7 ± 1.8	20.6 ± 0.6	0

[a]Activity without solvent was set as 100%.

[b]All Data are the mean ± SD (n = 3).

Table S4 Scores obtained from docking of EstM2 with di- and mono-substituted phthalate esters[a]

Ligand	FlexX Output			Autodock output	
	FlexX score	Clash score	Residues interacting with the ligand	Binding energy	Residues interacting with the ligand
Di-substituted phthalates					
Dimethyl phthalate (DMP)	-14.3309	1.6329	Ala67,Ser68, Phe128, Asp144, Met146, Tyr175, Thr247, Ile250, Trp343, Gly344, Gly345, Ala346, Met372	-6.02 (100%)	Ala67, Ser68,Lys71,Tyr126,Phe128,Asp144,Met146,Tyr175, Pro243,Thr247,Ile250, Ala268, Trp343, Gly344, Gly345, Ala346, Met372
Diethyl phthalate (DEP)	-13.2594	1.7830	Ala67, Ser68, Phe128,Asp144, Met146, Tyr175,Thr247, Ile250, Ala268, Glu313, Trp343, Gly344, Gly345, Ala346, Met372	-6.69 (90%)	Ala67, Ser68, Lys71, Tyr126, Phe128, Asp144, Met146,Tyr175, Pro243, Thr247, Ile250, Gly267, Ala268, Glu313, Trp343, Gly344, Gly345, Ala346, Met372
Di-*n*-butyl phthalate (DBP)	-7.8023	2.9395	Ser68, Phe128, Leu129, Trp173, Leu174, Tyr175, Pro243, Gly246, Thr247, Val248, Ile250, Pro312, Glu313, Trp343, Gly344, Gly345, Ala346, Tyr369, Met372.	-7.92 (56%)	Ala67, Ser68,Lys71,Tyr126,Phe128,Asp144, Gly145, Met146,Tyr175,Pro243,Thr247, Ile250,Ala268,Glu313,Trp343, Gly344, Gly345, Ala346,Met372
Butyl benzyl phthalate (BBP)	-14.8263	3.9856	Ser68, Phe128, Met146, Trp173, Tyr175, Pro243,Gly246,Thr247, Ile250, Pro312,Glu313, Trp343, Gly344, Gly345,Ala346, Met372	-8.51 (42%)	Ala67,Ser68,Lys71,Tyr126,Phe128,Asp144,Gly145,Tyr175,Pro243,Thr247,Ile250, Gly267, Ala268,Glu313,Trp343, Gly344, Gly345, Ala346,Met372, Asp373.
Diphenyl phthalate (DPP)	-14.7416	4.9278	Ser68, Phe128, Met146, Trp173,Tyr175, Pro243,Gly246,Thr247, Val248, Ile250, Pro312, Glu313, Trp343,Gly344, Gly345, Ala346, Met372	-9.98 (98%)	Ala67,Ser68,Tyr126,Phe128,Asp144,Met146, Tyr175,Ile250,Gly267, Ala268,Glu313,Trp343, Gly344, Gly345, Ala346,Met372.
Di-*n*-octyl phthalate	ND	ND	ND	-8.6 (32%)	Ser68,Tyr126,Phe128,Asp144, Met146,Tyr175,Pro243,Thr247,

(DOP)[b]					Val248,Gly249, Ile250,Ala268, Glu313, Trp343, Gly344, Gly345, Ala346, Tyr369, Met372.
Di-2-Ethyl hexyl phthalate (DEHP)[b]	-14.6999	6.0994	Ala67, Ser68, Tyr126, Phe128, Met146, Tyr175, Pro243,Thr247, Val248,Gly249, Ala268, Glu313, Trp343, Gly344, Gly345, Ala346, Tyr369, Met372	-9.09 (28%)	Ala67,Ser68,Tyr126,Phe128,Asp144,Met146,Tyr175,Pro243,Thr247,Val248, Gly249, Ile250,Ile250,Ala268,Glu313,Trp343, Gly344, Gly345, Ala346,Tyr369,Met372
Mono-substituted phthalates					
Monomethyl phthalate (MMP)	-11.9558	0.2836	Ala67, Ser68, Phe128,Thr247,Ile250, Pro312, Glu313, Trp343,Gly344, Gly345, Ala346, Met372	-4.89 (96%)	Ala67, Ser68,Met146,Tyr175,Ile250,Trp343, Gly344, Gly345, Ala346,Met372
Monoethyl phthalate (MEP)	-12.2165	0.6920	Ser68, Phe128, Tyr175, Pro243, Thr247,Ile250, Glu313, Trp343, Gly344, Gly345, Ala346, Met372	-5.37 (90%)	Ala67, Ser68,Phe128,Met146,Tyr175,Pro243,Thr247,Ile250, Gly267, Ala268, Glu313, Trp343, Gly344, Gly345, Ala346,Met372
Monobenzyl phthalate (MBzP)	-17.6710	3.0616	Ala67, Ser68, Tyr126, Phe128, Asp144, Met146, Trp173, Tyr175, Pro243, Thr247, Ile250, Ala268, Pro312, Glu313, Trp343, Gly344, Gly345, Ala346, Met372	-5.78 (56%)	Ala67, Ser68,Phe128,Met146,Tyr175,Pro243,Thr247, Val248, Ile250,Glu313,Trp343, Gly344, Gly345, Ala346, Met372
Monophenyl phthalate (MPP)	-15.9153	2.1186	Ala67,Ser68,Tyr126, Phe128, Tyr175, Pro243, Thr247, Ile250, Trp343, Gly344, Gly345, Ala346, Met372	-6.86 (50%)	Ala67, Ser68,Tyr126,Asp144,Met146, Tyr175,Ile250, Gly267, Ala268, Trp343, Gly344, Gly345, Ala346, Met372

[a] Arranged in order of descending rates of hydrolysis

[b] Not hydrolyzed by EstM2

ND, docking not detected

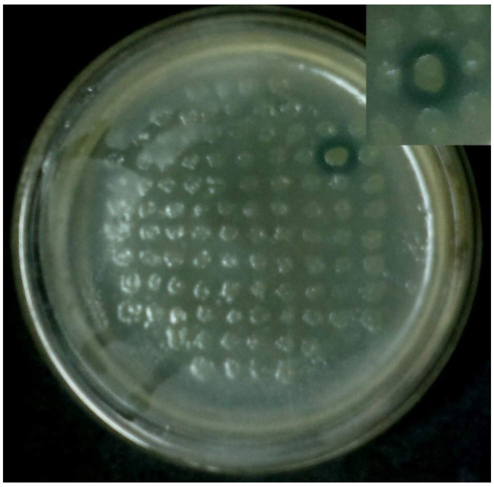

Fig. S1 Screening for lipolytic enzyme activity on 1% (v/v) tributyrin agar plate. Inset shows formation of clear zone by fosmid clone, *E. coli* EPI300-fosEstM2

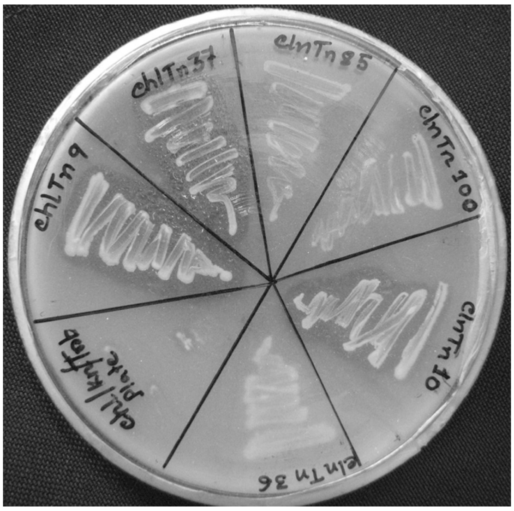

Fig. S2 Mutants of esterase-positive clone by *in vitro* transposon mutagenesis using the commercially available EZ-Tn*5* <KAN-2> insertion kit (Epicentre, USA). Mutants did not exhibit zone formation on tributyrin agar plate.

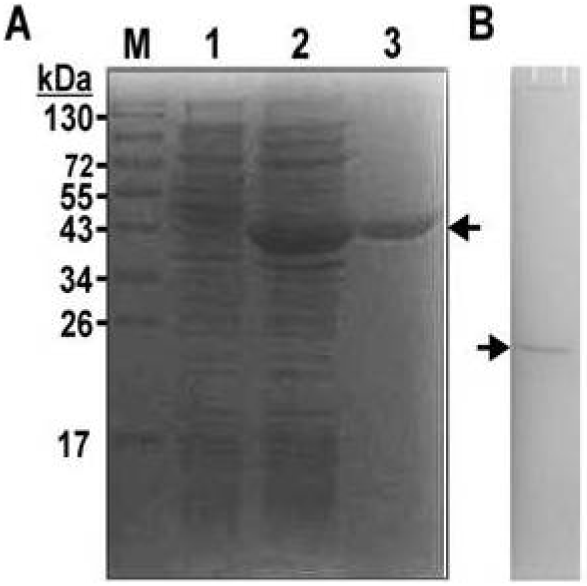

Fig. S3 (A)SDS-PAGE of the purified EstM2 protein. M, molecular size markers; lane 1, whole-cell extracts before induction; lane 2, whole-cell extracts after induction; lane 3, EstM2 purified by Ni-nitrilotriacetic acid column (denatured); **(B)** Activity staining of EstM2 purified by Ni-nitrilotriacetic acid column (non-denatured). Arrows indicate position of the band corresponding to EstM2.

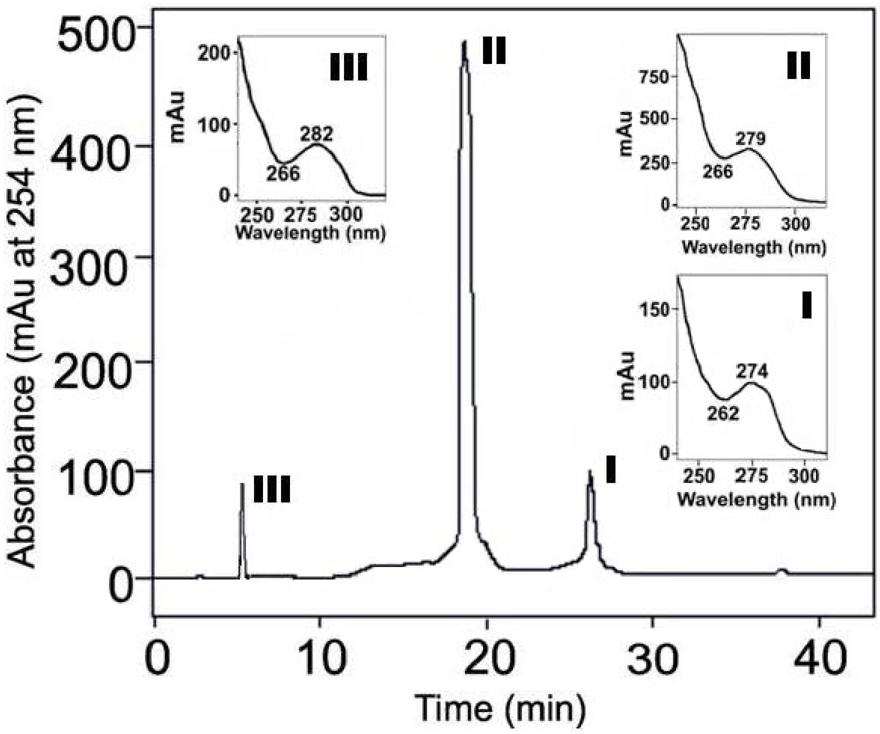

Fig. S4 HPLC profile of di-*n*-butyl phthalate and its metabolic intermediates in the reaction mixture containing 1 mM of substrate (dissolved in methanol) and 0.25 μg of purified protein (EstM2) in a final volume of 1 ml Tris-HCl buffer (50 mM, pH 8.0), incubated for 1 h. Insets, UV-visible spectra of peaks obtained with diode array analysis. I, di-*n*-butyl phthalate; II, mono-*n*-butyl phthalate, III, phthalic acid.
